# A synergistic effect of secondhand smoke with vitamin D deficiency on cognitive impairment in older adults: a cross sectional study

**DOI:** 10.3389/fnut.2025.1533193

**Published:** 2025-02-11

**Authors:** Yan Li, Qianqian Shen, Chengyu Chen, Xueru Yin, Xinru Wang, Xiyue Yang, Xueqian Zhang, Lei Chen, Jian Xu, Guifang Gong

**Affiliations:** ^1^School of Public Health, Qingdao University, Qingdao, China; ^2^School of Public Health, Peking University, Beijing, China; ^3^Department of Elderly Health Management, Shenzhen Center for Chronic Disease Control, Shenzhen, China; ^4^Qingdao Municipal Hospital, Qingdao, China

**Keywords:** 25-hydroxyvitamin D, secondhand smoke, cognitive impairment, elderly, synergistic effect

## Abstract

**Objectives:**

To investigate whether exposure to secondhand smoke (SHS) aggravates the detrimental effect of vitamin D deficiency (VDD) on cognitive performance in the elderly.

**Methods:**

Based on National Health and Nutrition Examination Surveys (NHANES) 2011–2014, 1,446 non-smoking participants (≥ 60 years old) with detailed serum 25-hydroxyvitamin D [25(OH)D], concentration of cotinine and tests score of cognitive function were included. Cognitive impairment was defined as having a cognitive score in the lowest quartile. The possible synergistic effect of SHS with VDD on cognitive impairment was evaluated by using a multivariable logistic regression model.

**Results:**

VDD was independently associated with risk of low the Digit Symbol Substitution Test (DSST) scores, increased by nearly 60% [< 34, adjusted odds ratio (aOR) = 1.62, 95% CI: 1.03 ~ 2.53]. Although it only had an association with cognitive impairment indicated by DSST and the Animal Fluency test (AFT) in the crude model, SHS exposure showed significant synergistic effects with VDD on DSST (aOR: 3.03, 95% CI: 1.57 ~ 5.83, P_interaction_ = 0.001) and AFT (aOR: 2.40, 95% CI: 1.34 ~ 4.29, P_interaction_ = 0.003), respectively, after adjusting for the possible confounders. In further stratified analysis, a more obvious synergistic effect of SHS with VDD on DSST (aOR: 4.73, 95%CI:1.77 ~ 12.68, P_interaction_ = 0.002) and AFT (aOR: 5.30, 95%CI: 1.63 ~ 17.24, P_interaction_ = 0.006) was found in obese and overweight subjects, respectively.

**Conclusion:**

SHS exposure had synergistic effect with VDD on cognitive impairment among elderly and the interaction effect was more obvious in overweight and obese individuals.

## 1 Introduction

With increasing age of the global population, age-related cognitive decline and the correspondent strategy to cope with it has become an intensive interesting topic in scientific research. Studies based on population data indicate that about one-third of Alzheimer’s disease (AD) cases worldwide could be attributed to factors that can potentially be modified, including nutrition and environmental harmful substance exposure (1, 2). Vitamin D deficiency (VDD), a worldwide concern, has been suggested to be associated with cognitive impairment, particularly among older people (3). A meta-analysis involving 7,688 subjects shown a significantly increased risk of cognitive impairment in VDD subjects (4). Similarly, serum Vitamin D(VD) levels were positively correlated with cognitive performance in the United States elderly (5, 6). Cohort studies have demonstrated that severe VDD is associated with about 2-fold increased risk of developing AD (7). Other than that, VD supplementation has been shown to improve cognitive impairment both in mild cognitive impairment (MCI) elderly (8) and AD patients (9) in randomized controlled trials, although some trials did not find the same effects (10).

There is an increasing amount of evidence indicating that the use of tobacco, both through active smoking and exposure to secondhand smoke (SHS), is linked to a decline in cognitive abilities (11, 12), as well as accelerates cognitive decline (13) and dementia (14). Despite a decrease in cigarette smoking rates in the United States, the prevalence of smoking among adults still stood at 19.0% in 2020 (15). As far as we know, older individuals who spend more time indoors are at a higher risk of being exposed to SHS, which has been reported to elevate the risk of cognitive decline including reduced processing speed and executive function (16). Mounting evidence support the correlation between VDD and cognitive impairment. Mechanistically, SHS in related to VDD and cognitive impairment. A few studies have shown that tobacco has an endocrine-disrupting effect and has linked it to dysfunctional VD endocrine systems accompanied with declined serum levels of VD metabolites (17–19). VD-parathyroid hormone (PTH) axis dysfunction due to tobacco smoke exposure may lead to disruption of VD metabolism and dysregulation of VD metabolism-related enzyme genes (20). However, till now, whether SHS is involved in the relationship between VDD and cognitive impairment has not been reported.

Taken together, we hypothesized that there could be a synergistic effect of SHS with VDD on cognitive decline in older individuals. Therefore, we examined how the simultaneous exposure to SHS and VDD affects cognitive function in people aged 60 and above, evaluating the potential interaction effects by using data from National Health and Nutrition Examination Surveys (NHANES) 2011–2014. Understanding this relationship could assist health authorities in making informed decisions about implementing health promotion strategies and interventions to prevent cognitive impairment in the elderly.

## 2 Methods

### 2.1 Study population

**Figure 1:**
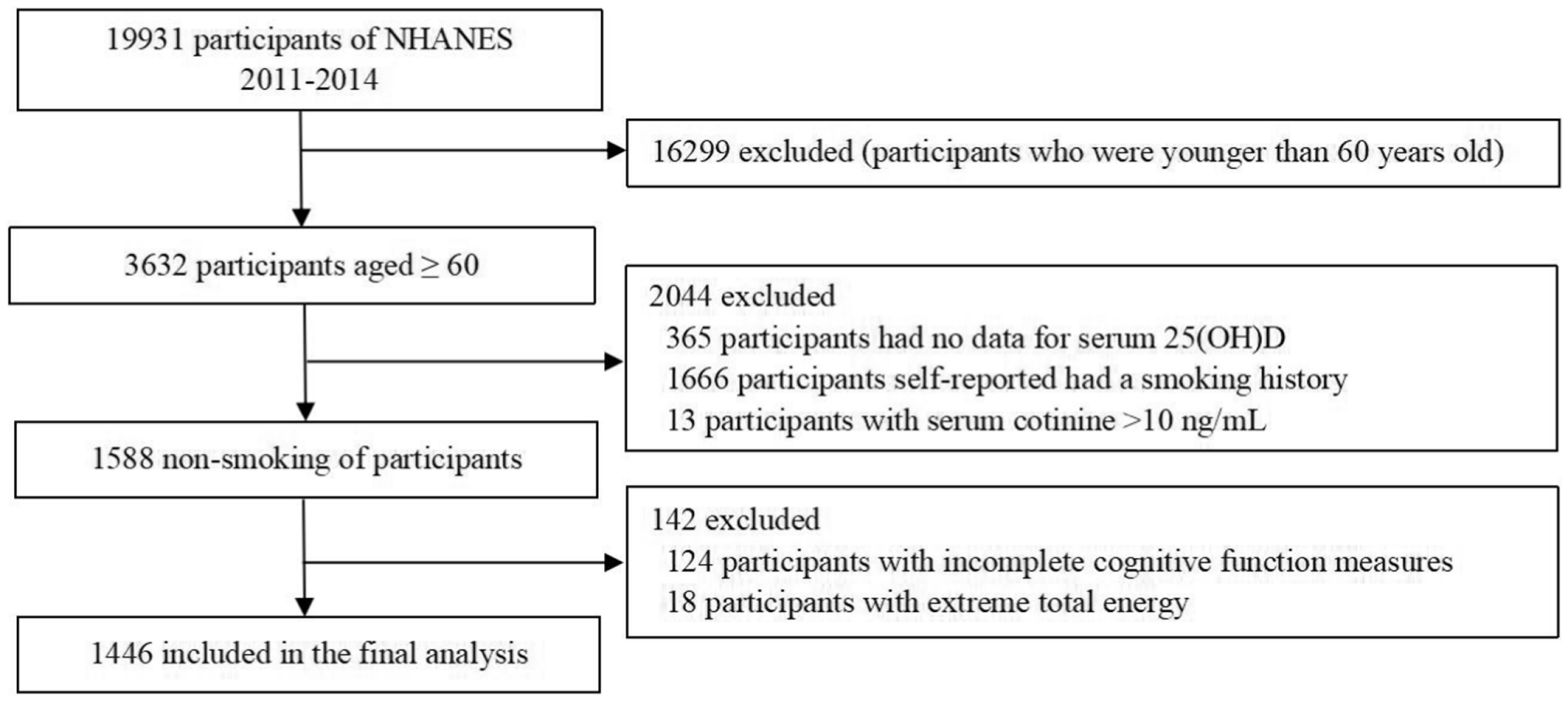
Flow chart of the screening process for the eligible participants.

Data from NHANES 2011–2014 with cognitive tests in the elderly were utilized. NHANES is conducted by the Centers for Disease Control and Prevention’s National Center for Health Statistics (NCHS) to assess the health and nutritional status of the United States civilian population via interviews and physical exams. Conducted every 2 years since 1999, participants represented the general population of the United States through a complex multi-stage sampling method. The first was the conduct of a questionnaire, followed by a physical examination and the collection of biological specimens of the participants. Participants with detailed serum 25-hydroxyvitamin D [25(OH)D], cotinine data, and cognitive performance test scores were included in the analysis (*N* = 19,931). Exclusion criteria included younger than 60 years old (*n* = 16,299); missing data for serum 25(OH)D (*n* = 365); self-reported active smoking history including chewing tobacco or other forms of snus (*n* = 1,666); serum cotinine >10 ng/mL (the cutoff value for active smoking) (n = 13); missing data for cognitive outcomes (*n* = 124); abnormal energy intake (daily energy intake >5,000 kcal or < 500 kcal, *n* = 18). Finally, we analyzed data from a total of 1,446 individuals who were elderly non-smokers (≥ 60 years old) taken from NHANES 2011–2014. The process of participant selection is shown in Figure 1.

### 2.2 Assessment of cognitive impairment

The tests for cognitive function in NHANES 2011–2014 include word learning and recall modules from the Consortium to Establish a Registry for Alzheimer’s disease (CERAD), the Animal Fluency test (AFT) and the Digit Symbol Substitution Test (DSST).

The CERAD consists of three consecutive learning trials and a delayed recall. During the learning trials, participants are given the instruction to pronounce 10 words that are not related to each other. They were required to remember as many of these words as possible right away. The maximum score for each trial is 10. The delayed word recall test occurred after completion of the other two cognitive exercises (AFT and DSST). Higher scores on this item indicate better cognitive performance.

Categorical verbal fluency in executive function was examined with the AFT, which has been demonstrated that scores can be used to differentiate individuals who have normal cognitive abilities from those with MCI and more severe cognitive impairments (21, 22). During the assessment, participants are given 1 min to list as many animals as they can. Each correctly named animal earns one point, and the total score is calculated by adding up all the correct answers. To familiarize participants with the task, they are initially asked to name three clothing items as a practice test. If a participant is unable to name three articles of clothing, they are not included in the subsequent assessment.

The DSST depends on speed of processing, sustained attention, and working memory. Participants are asked to fill in the blank boxes according to the symbols corresponding to the numbers and copy the corresponding symbols in the 133 boxes of adjacent numbers in 2 min. The score indicates the overall count of accurate matches.

Since the three tests mentioned above did not have established criteria for identifying low cognitive performance, we relied on the standards used in relevant published research (23). For each test, we determined the cutoff point by selecting the highest value among those in the lowest quartile. Participants who scored below these cutoff points were classified as having impaired cognitive performance, namely, < 20 for the CERAD, < 13 for the AFT, and < 34 for the DSST, respectively.

### 2.3 Serum 25(OH)D measurement

After fasted for 9 h, blood was drawn from the subjects processed by a mobile examination center (MEC) and immediately frozen at −30°C for further measurements. Ultra-performance liquid chromatography–tandem mass spectrometry (UHPLC–MS/MS) (24) quantitative assay was used to detect serum 25(OH)D, the optimal indicator of nutritional status of VD in the body. Quantification was done by comparing the unknown analyte’s peak area to the known amount of analyte in the calibrator solution. The calculation was corrected by comparing the unknown peak area with the peak area of the matching internal standard in the calibrator solution (25).

### 2.4 Assessment of SHS exposure

In this study, SHS was assessed using self-reported data by questionnaires combined with serum cotinine concentration. First of all, according to the answers to questions SMQ020 and SMQ040, adults surveyed responses to three questions (SMQ681, SMQ851, and SMQ863) about tobacco use (smoking and nicotine replacement therapy products) in the past 5 days to exclude active smokers. Next, if a participant reports exposure to SHS in the last 7 days, self-reported SHS is considered present. In case of missing questionnaires, SHS exposure was measured with serum cotinine and defined as exposed if the subject had a cotinine level of 0.05–10 ng/mL (26, 27). While those below 0.05 ng/mL are considered non-SHS exposed. Cotinine is determined by an isotope dilution-high performance liquid chromatography/atmospheric pressure chemical ionization tandem mass spectrometry (ID HPLC-APCI MS/MS) (28).

### 2.5 Covariates

We included factors of age, sex, race, educational level, marital status, poverty index (PIR), alcohol use, physical activity, body mass index (BMI), diabetes, stroke, asthma, and congestive heart failure as covariates. A computer-assisted personal interview was utilized to gather demographic information of the participants. Alcohol use (ALQ101 and ALQ110) and vigorous recreational activities (PAQ650) were defined based on self-reported data by questionnaires. We explored chronic conditions of diabetes, stroke, asthma, and congestive heart failure in the medical conditions questionnaire.

### 2.6 Statistical analysis

Serum 25(OH)D was treated as a categorical variable [sufficiency (≥ 75 nmol/L), insufficiency (50 ~ 75 nmol/L), and deficiency (< 50 nmol/L)] as suggested by the Institute of Medicine (IOM) (29). Depending on exposure to SHS, the participants were categorized into two groups (yes or no). Main confounders were categorized as follows: age (60 ~ 69 y, 70 ~ 79 y, and ≥ 80 y); sex (male and female); race (Mexican American, Non-Hispanic White and Black, and Other); education (high school and below/general equivalency diploma, some college/associates degrees, and college degree/above); marital status (married/living with a partner, never married/separated/widowed/divorced); PIR (< 1, and ≥ 1); BMI (< 25 kg/m^2^, 25 ~ 29.99 < kg/m^2^, and ≥ 30 kg/m^2^). In the interaction analysis, according to their nutritional status of VD and exposure to SHS, we categorized the entire population into six groups. Logistic regression models to examine the relationship between exposure to VDD and/or SHS and the prevalence of cognitive performance. Odds ratios [OR, with 95% confidence intervals (CI)] were employed to assess the magnitude of the effect. In multivariate logistic regressions, model 1 was adjusted for age, sex, race, education level, marital status, PIR, and model 2 further adjusted for alcohol use, physical activity, BMI, diabetes, stroke, asthma, and congestive heart failure. Although there were no significant differences in sex, BMI, and the presence of asthma between subjects with or without cognitive impairment (*p* > 0.05), we still adjusted them to minimize the potential residual confounding effects. We assessed the possible synergistic effects of SHS with VDD using the multiplicative scale method. Two-sided *p* values were calculated to evaluate the significance of each term in the logistic regression models to compare the OR of SHS on cognitive function in different groups based on levels of serum 25(OH)D. In order to further elucidate the associations, we conducted additional analysis by stratifying the data according to age, sex, and BMI. Considering that stroke made up only 5.7% of the participants, we performed sensitivity analysis by excluding stroke and compared the results. The statistical analysis was conducted using SPSS 22.0 software, and a significance level of *p* < 0.05 was considered statistically significant.

### 2.7 Ethics approval and consent to participate

The research was carried out in accordance with the principles outlined in the Declaration of Helsinki. Participants in this study are all in accordance with the study ethics guidelines and have informed consent.

## 3 Results

The characteristics of all participants included are shown in Table 1 Individuals with low cognitive performance in all the three tests were more likely to be older, other Hispanic and other Racial, low educated, unmarried (or not living with a partner), less physically active, poverty stricken, with higher prevalence of diabetes, stroke, and heart failure, and less proportion of alcohol drinks (*p* < 0.01). Individuals with impaired cognitive performance had relatively lower serum 25(OH)D levels in all tests, and they had higher prevalence of SHS exposure in the AFT and DSST (*p* < 0.05). Table 2 shows the effects of exposure to VDD, SHS on cognitive impairment. In binary logistic regression analyses, compared to those with sufficient VD, subjects with VDD had 62% increased odds of cognitive impairment indicated by DSST after adjusting for the possible confounders in the final model [adjusted odds ratio (aOR): 1.62; 95% CI: 1.03, 2.53]. In the AFT, a significant correlation was observed in VDD with impaired cognitive performance in the crude analysis (OR: 1.47; 95% CI: 1.06, 2.04), and a marginal significant correlation remained after adjusting for age, sex, race, education, marital status and PIR (aOR: 1.41; 95% CI: 0.97, 2.05, *p* = 0.072). In the CERAD, a marginal significant correlation was observed in VDD with impaired cognitive performance in the crude analysis (OR: 1.38; 95% CI: 0.97, 1.95, *p* = 0.071) while no significant correlations remained after adjusting for the possible confounding factors, while a statistical significant correlation remained in insufficient VD in the final multivariable model (aOR: 1.41; 95% CI: 1.01, 1.97). Exposure to SHS was associated with cognitive impairment both in the DSST (OR: 1.40; 95% CI: 1.06, 1.85) and the AFT (OR: 1.41; 95% CI: 1.08, 1.86) in the crude models. After adjusting for age, sex, race, education, marital status and PIR, only a marginal statistical significance was observed in the AFT (aOR: 1.31; 95% CI: 0.96, 1.78, *p* = 0.088).

**Table 1:** Characteristics of the subjects by cognitive impairment, NHANES 2011–2014 [*N* = 1,446, *n* (%)].

Characteristics	CERAD			AFT			DSST		
	Normal Cognitive Performance	Impaired Cognitive Performance	*p*-value	Normal Cognitive Performance	Impaired Cognitive Performance	P-value	Normal Cognitive Performance	Impaired Cognitive Performance	P-value
Number of subjects	1,093 (75.6)	322 (22.3)		1,067 (73.8)	348 (24.1)		1,040 (71.9)	329 (22.8)	
Age, years			<0.001			<0.001			<0.001
60~	613 (56.1)	103 (32.0)		589 (55.2)	129 (37.1)		596 (57.3)	119 (36.2)	
70~	319 (29.2)	101 (31.4)		303 (28.4)	116 (33.3)		290 (27.9)	110 (33.4)	
≥ 80 years	161 (14.7)	118 (36.6)		175 (16.4)	103 (29.6)		154 (14.8)	100 (30.4)	
Sex (%)			<0.001			0.334			0.431
Male	355 (32.5)	144 (44.7)		383 (35.9)	115 (33.0)		364 (35.0)	123 (37.4)	
Female	738 (67.5)	178 (55.3)		684 (64.1)	233 (67.0)		676 (65.0)	206 (62.6)	
Race (%)			0.407			0.020			<0.001
Mexican America	97 (8.9)	36 (11.2)		100 (9.4)	31 (8.9)		70 (6.7)	49 (14.9)	
Non-Hispanic White and Non-Hispanic Black	739 (67.6)	208 (64.6)		735 (68.9)	216 (62.1)		741 (71.2)	185 (56.2)	
Other Hispanic and Other Race	257 (23.5)	78 (24.2)		232 (21.7)	101 (29.0)		229 (22.0)	95 (28.9)	
Education level (%)			<0.001			<0.001			<0.001
College degree or above	330 (30.2)	54 (16.8)		332 (31.1)	54 (15.5)		351 (33.8)	33 (10.0)	
Some college/associates degrees	324 (29.6)	60 (18.6)		307 (28.8)	75 (21.6)		323 (31.1)	52 (15.8)	
High school and below/general equivalency diploma	438 (40.1)	208 (64.6)		428 (40.1)	218 (62.6)		366 (35.2)	243 (73.9)	
Marital status (%)			0.002			0.002			<0.001
Married/living with partner	653 (59.7)	161 (50)		638 (59.8)	175 (50.3)		639 (61.4)	160 (48.6)	
Never married/separated/widowed/divorced	438 (40.1)	160 (49.7)		428 (40.1)	171 (49.1)		399 (38.4)	169 (51.4)	
Family PIR (%)			<0.001			<0.001			<0.001
PIR ≥ 1	885 (78.2)	224 (69.6)		856 (80.2)	226 (64.9)		857 (90.1)	199 (60.5)	
PIR < 1	135 (12.4)	74 (23.0)		120 (11.2)	85 (24.4)		94 (9.9)	100 (30.4)	
BMI, kg/m^2^			0.196			0.756			0.677
< 25	286 (26.2)	90 (28.0)		279 (26.1)	94 (27.0)		281 (27.0)	82 (24.9)	
25 ~ 30	368 (33.7)	116 (36.2)		378 (35.4)	113 (32.5)		357 (34.3)	118 (35.9)	
≥ 30	429 (39.2)	106 (32.9)		403 (37.8)	128 (36.8)		396 (38.1)	117 (35.6)	
Diabetes (%)	217 (19.9)	87 (27.0)	0.006	199 (18.7)	102 (29.3)	<0.001	187 (18.0)	95 (28.9)	<0.001
Stoke (%)	50 (4.6)	31 (9.6)	0.001	49 (4.6)	31 (8.9)	0.002	41 (3.9)	33 (10.0)	<0.001
Asthma (%)	158 (14.5)	31 (9.6)	0.026	148 (13.9)	41 (11.8)	0.326	136 (13.1)	45 (13.7)	0.788
Congestive heart failure (%)	51 (4.7)	42 (13.0)	<0.001	55 (5.2)	37 (10.6)	<0.001	44 (4.2)	42 (12.8)	<0.001
Alcohol drinking (%)	558 (51.1)	137 (42.5)	0.022	560 (52.5)	134 (38.5)	<0.001	567 (54.5)	119 (36.2)	<0.001
Recreational Physical activity (%)	126 (11.5)	14 (4.3)	<0.001	122 (11.4)	20 (5.7)	0.002	132 (12.7)	6 (1.8)	<0.001
25(OH)D_2_ + 25(OH)D_3_(%)			0.001			0.056			0.010
≥ 75 nmol/L	576 (52.7)	134 (41.6)		548 (51.4)	165 (47.4)		550 (52.9)	148 (45.0)	
50 ~ 75 nmol/L	333 (30.5)	129 (40.1)		354 (33.2)	110 (31.6)		331 (31.8)	110 (33.4)	
< 50 nmol/L	184 (16.8)	59 (18.3)		165 (15.5)	73 (21.0)		159 (15.3)	71 (21.6)	
SHS (%)	266 (24.3)	75 (23.3)	0.700	242 (22.7)	102 (29.3)	0.012	234 (22.5)	95 (28.9)	0.018

**Table 2:** Odds ratios of cognitive impairment according to serum 25(OH)D concentrations and SHS as categorical using a logistics regression model, NHANES 2011–2014 (*N* = 1,446).

			CERAD						AFT			
	Crude model OR (95% CI)	*p*- value	Model 1 OR (95% CI)	*p*- value	Model 2 OR (95% CI)	*p*- value	Crude model OR (95% CI)	*p*- value	Model 1 OR (95% CI)	*p*- value	Model 2 OR (95% CI)	*p*- value
25(OH)D level												
<50 nmol/L	1.38 (0.97, 1.95)^#^	0.071	1.18 (0.79, 1.77)	0.423	1.10 (0.72, 1.70)	0.658	1.47 (1.06, 2.04)*	0.021	1.41 (0.97, 2.05)^#^	0.072	1.30 (0.87, 1.93)	0.202
50–75 nmol/L	1.67 (1.26, 2.20)*	<0.001	1.53 (1.11, 2.10)*	0.010	1.41 (1.01, 1.97)*	0.047	1.03 (0.78, 1.36)	0.823	0.99 (0.72, 1.36)	0.957	0.99 (0.71, 1.37)	0.926
≥75 nmol/L	Ref.	—	Ref.	—	Ref.	—	Ref.	—	Ref.	—	Ref.	—
SHS												
Yes	0.94 (0.70, 1.27)	0.700	0.88 (0.63, 1.23)	0.446	0.88 (0.62, 1.25)	0.746	1.41 (1.08, 1.86)*	0.013	1.31 (0.96, 1.78)^#^	0.088	1.24 (0.90, 1.72)	0.189
No	Ref.	—	Ref.	—	Ref.	—	Ref.	—	Ref.	—	Ref.	—

				DSST		
	Crude model OR (95% CI)		Model 1 OR (95% CI)		Model 2 OR (95% CI)	
25(OH)D level						
<50 nmol/L	1.66 (1.19, 2.32)*	0.003	1.69 (1.11, 2.56)*	0.014	1.62 (1.03, 2.53)*	0.036
50–75 nmol/L	1.24 (0.93, 1.64)	0.142	1.11 (0.79, 1.57)	0.547	1.13 (0.78, 1.64)	0.525
≥75 nmol/L	Ref.		Ref.		Ref.	
SHS						
Yes	1.40 (1.06, 1.85)*	0.019	1.27 (0.90, 1.78)	0.174	1.17 (0.81, 1.68)	0.405
No	Ref.		Ref.		Ref.	

Model 1: adjusted for age, sex, race, education level, marital status, PIR. Model 2: Model 1+ alcohol use, physical activity, BMI, diabetes, stroke, asthma, and congestive heart failure. *Significant at *p* < 0.05; #Borderline significant, 0.05 < *p* < 0.1.

The results of the interaction analysis of VDD and SHS on cognitive impairment was shown in Table 3. Compared with persons with sufficient VD level and non-SHS exposure, with VDD and SHS exposurers had significantly higher odds of cognitive impairment in the DSST (aOR: 3.03; 95% CI: 1.57, 5.83) and the AFT (aOR: 2.40; 95% CI: 1.34, 4.29) after adjusting for the possible confounders.

**Table 3:** Interaction analysis of SHS with VD status on the risk of cognitive impairment in the elderly, NHANES 2011–2014 (*N* = 1,446).

			CERAD						AFT			
	Crude Model OR (95% CI)	*p*- value	Model 1 OR (95% CI)	*p*- value	Model 2 OR (95% CI)	*p*- value	Crude Model OR (95% CI)	*p*- value	Model 1 OR (95% CI)	*p*- value	Model 2 OR (95% CI)	*p*- value
Deficiency *SHS	1.42 (0.82, 2.47)	0.209	1.37 (0.72, 2.60)	0.328	1.23 (0.62, 2.44)	0.556	2.21 (1.35, 3.61)*	0.002	2.59 (1.49, 4.49)*	<0.001	2.40 (1.34, 4.29)*	0.003
Insufficiency *SHS	1.54 (0.98, 2.41)	0.061	1.28 (0.76, 2.16)	0.358	1.28 (0.75, 2.20)	0.366	1.38 (0.89, 2.13)	0.154	1.08 (0.65, 1.79)	0.780	0.98 (0.57, 1.69)	0.953
Sufficiency *SHS	0.73 (0.44, 1.20)	0.218	0.66 (0.38, 1.12)	0.125	0.59 (0.34, 1.05)	0.073	1.15 (0.75, 1.76)	0.519	0.97 (0.60,1.57)	0.910	0.94 (0.57, 1.55)	0.794
Deficiency *non-SHS	1.25 (0.82, 1.88)	0.300	0.96 (0.59, 1.55)	0.858	0.89 (0.54, 1.49)	0.663	1.23 (0.82, 1.84)	0.320	0.98 (0.61, 1.57)	0.928	0.88 (0.53, 1.45)	0.605
Insufficiency*non-SHS	1.58 (1.16, 2.17)*	0.004	1.44 (1.00, 2.07)*	0.049	1.25 (0.85, 1.84)	0.251	0.96 (0.70, 1.33)	0.806	0.95 (0.66, 1.37)	0.802	0.96 (0.66, 1.41)	0.838
Sufficiency *non-SHS	Ref.	—	Ref.	—	Ref.	—	Ref.	—	Ref.	—	Ref.	—

			DSST			
	Crude Model OR (95% CI)	*p*- value	Model 1 OR (95% CI)	*p*- value	Model 2 OR (95% CI)	*p*- value
Deficiency *SHS	2.49 (1.50, 4.11)*	<0.001	3.25(1.76, 6.00)*	<0.001	3.03(1.57, 5.83)*	0.001
Insufficiency *SHS	1.57 (1.00, 2.48)	0.051	1.11(0.64, 1.95)	0.710	1.06(0.59, 1.93)	0.839
Sufficiency *SHS	1.13 (0.72, 1.76)	0.593	0.88(0.51, 1.50)	0.633	0.74(0.42, 1.33)	0.316
Deficiency *non-SHS	1.38 (0.92, 2.09)	0.123	1.10(0.65, 1.84)	0.732	1.02(0.59, 1.77)	0.948
Insufficiency*non-SHS	1.17 (0.84, 1.62)	0.350	1.07(0.72, 1.60)	0.729	1.06(0.69, 1.62)	0.807
Sufficiency *non-SHS	Ref.		Ref.	—	Ref.	—

Model 1: adjusted for age, sex, race, education level, marital status, PIR. Model 2: Model 1+ alcohol use, physical activity, BMI, diabetes, stroke, asthma, and congestive heart failure. *Significant at *p* < 0.05.

Furthermore, considering that participants with a history of stroke made up only 5.7% of the total participants, we performed sensitivity analysis by excluding them. In the fully adjusted model, the negative associations remained, and the size effect was larger in DSST. Hence, sensitivity analysis proves that the regression model is robust as shown in the Supplementary material S1.

**Figure 2:**
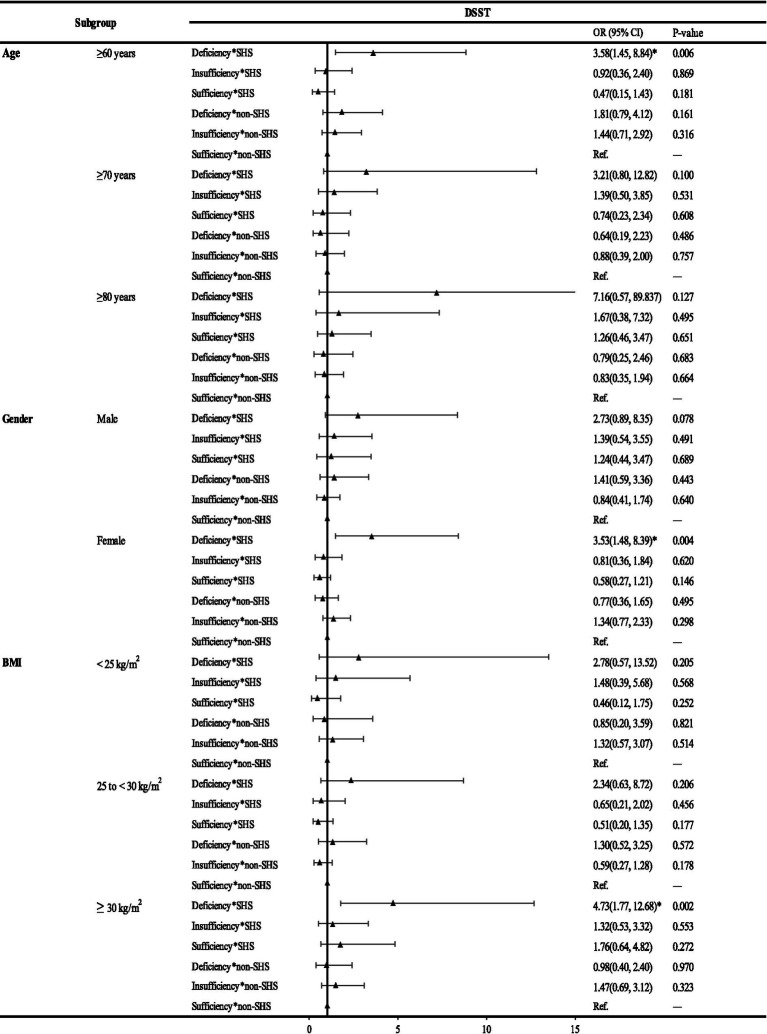
Subgroup analysis of the interaction of 25(OH)D and SHS on the risk of DSST in the elderly, NHANES 2011–2014 (*N* = 1,446). *: significant at *p* < 0.05. Abbreviations: DSST, the Digit Symbol Substitution Test; SHS, secondhand smoke; BMI, body mass index.

The results of subgroup analysis of the interaction effects of VDD and SHS on cognitive impairment were shown in Figure 2; Supplementary material S1. The interaction effects on cognitive impairment remained in those aged 60 ~ 69. Sex stratified analysis showed similar results in male and female as the total population. Male may be more sensitive to combined VDD and SHS exposure in the AFT than female (aOR: 3.10; 95% CI: 1.13, 8.49 vs. aOR: 2.21; 95% CI: 1.06, 4.63), while female may be more sensitive than male in DSST (aOR: 3.53; 95% CI: 1.48, 8.39 vs. aOR: 2.73; 95% CI: 0.89, 8.35, *p* = 0.078). In BMI stratified analysis, no synergistic effects of SHS with VDD on cognitive impairment indicated by all the three tests were observed in those of underweight or normal weight (BMI < 25 kg/m^2^). Compared to the total subjects, a more significant synergistic effect was observed in AFT (aOR: 5.30; 95% CI: 1.63, 17.24 vs. aOR: 2.40; 95% CI:1.34, 4.29) in overweight individuals (25 ≤ BMI < 30). For DSST, no synergistic effect remained in overweight elderly, while in obese individuals, we found a more significant synergistic effect (aOR: 4.73; 95% CI: 1.77, 12.68 vs. aOR: 3.03; 95% CI: 1.57, 5.83; Figure 2).

## 4 Discussion

Utilizing data from two continuous NHANES cycles of 1,446 eligible subjects, we found that SHS exposure had a significant synergistic effect with VDD on cognitive impairment in older adults, and the effect was more pronounced among overweight and obese participants. To our best knowledge, this is the first study of its kind which may provide important evidence to the control of cognitive impairment in older adults.

In accordence with previous studies, we found VDD was independently associated with cognitive impairment in older adults (30). VD has been reported to be involved in neuronal proliferation (31) with neuroprotective effects (32), and VDD was revealed to play an important role in the pathogenesis of dementia (33). Mechanistically, VD can have an impact on neurocognition through a variety of mechanisms, such as inducing neuroprotection, regulating calcium homeostasis, and regulating oxidative stress (34). VDD leads to dysregulations of perineuronal nets (35) and matrix metalloproteinases (36) and further contributes to cognitive decline and impairment. Older people are prior to have VDD mainly due to their reducing outdoor activities, as well as the decreasing bioavailability of VD in the body. There are differences in the results observed by different cognitive function tests in this study, which may be that the design of the test itself influenced the assessment results. Previous studies on cognition have also found more positive results for DSST and AFT than for CERAD, and this study also confirms this (37, 38). Specifically, VDD was reported to be associated with reduced processing speed and decreased verbal fluency (39), and a longitudinal study of Australian women found that individuals with VD > 25 nmol/L had better verbal fluency performance but was not associated with the CERAD (40). Consistently, we found similar effects of VDD on the special impaired cognitive performance verbal fluency in the total subjects as well as in the sex and BMI stratified analysis.

SHS exposure may increase the risk of overall cognitive impairment in older adults. As reported, Subjects exposed to SHS had a greater decline in memory scores (41). A cohort study of 2087 non-smoking older adults in Spain found SHS exposure was related to increased risk of overall cognitive impairment with decreased working memory capacity (42). Similarly, in a longitudinal study of 6,875 Chinese women, those who lived with smoking husbands had significantly faster declines in global cognitive function (43). However, in a cohort of 970 older people, Barnes et al. did not find a relationship between SHS and the risk of dementia (44). Smoking leads to cognitive dysfunction by destroying subcortical gray matter, the frontotemporal cortex(functions of language and movement), and the medial temporal lobe pathway (functions of language, memory, and mental activity) (45). In our study, in crude analysis, exposure to SHS was associated with nearly 40% higher risk of cognitive impairment both in the DSST and the AFT. However, after adjusting for the potential confounders, no significant associations remained. It may mainly be due to different criteria for judging SHS exposure (46) in these studies and more confirmative cohort researches are needed in the future.

**Figure 3:**
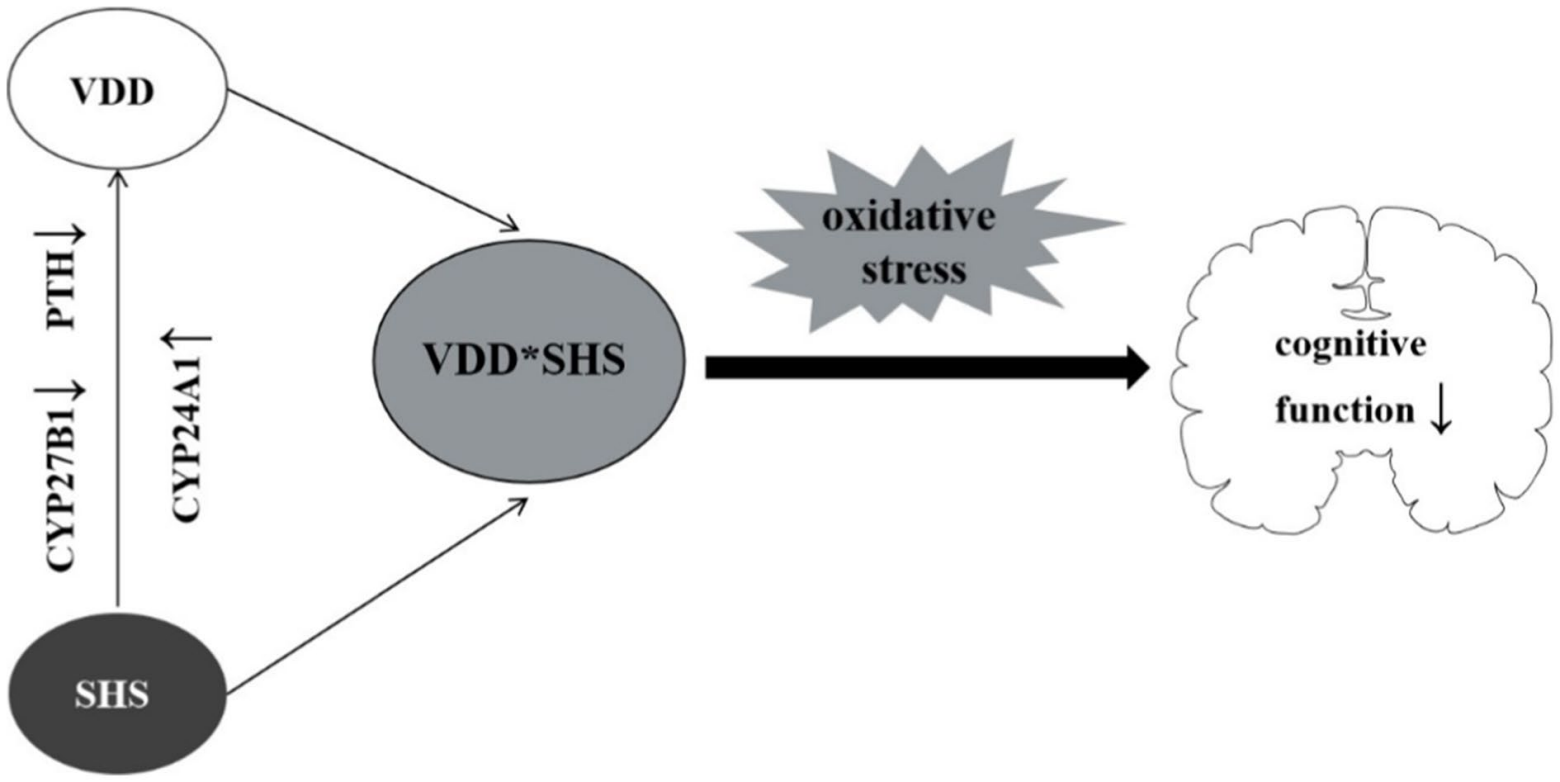
The latent mechanism.

It is worth noting that we found SHS has a synergistic effect with VDD on cognitive impairment in the elderly. Our novel findings are plausible concerning the biological mechanisms. On one hand, SHS may share the similar biological pathway leading to cognitive impairment as VDD. Previous studies found that cigarette smoke induced dysregulation of the balance between oxidants and antioxidants, inflammasome activation, reactive oxygen species (ROS) (47) production, and Ca^2+^ influx (48), finally leading to oxidative stress (49) in the body. Likewise, as reported, VDD was accompanied by increased ROS generation and intracellular free Ca^2+^ in brain nerve terminals (50), causing oxidative stress to weaken neuroprotective effects further (8). On the other hand, SHS is associated with VDD and cognitive impairment. Tobacco itself may cause VDD by inhibiting some of the key metabolic sites of VD. Smoking inhibits the expression of CYP27B1 (51) (the key enzyme required for activation of VD), reduces serum PTH level, increases the expression of CYP24A1 (52) (the key enzyme required for breakdown of VD), thereby reducing serum VD levels and cognitive impairment (53) (Figure 3). Other than that, smoking decreases VD intake from diet, calcium absorption, and reduces the cutaneous production of VD through skin aging (52).

Moreover, we found the synergistic effect of VDD with SHS on cognitive impairment was markedly more significant in overweight or obese elderly. Obesity is widely recognized as a chronic, low-grade inflammatory state (54). It has been reported that overweight and obese persons had impaired performance on memory (55), cognitive flexibility, and executive function (56). It has been reported that obese persons have lower temporal lobe volume due to brain atrophy (57, 58), which is in accordance with the impaired cognitive function. Similarly, exposure to SHS increases the inflammatory response (59), and oxidative stress in obese subjects (60). Therefore, this may partially explain the increased synergistic effects of SHS with VDD on cognitive impairment in overweight and obese elder persons. In addition, people with overweight and obese are more likely to develop VDD, mainly due to the less sun exposure than lean counterparts (61); VD levels are reduced due to volume dilution (62).

There are several strengths in our study. To begin with, our research makes use of a significant portion of the United States population as subjects and examines the impact of SHS with VDD on the deterioration of cognitive abilities. In addition, this study is the first of its kind to examine the connection between the combined exposure of VDD and SHS and the risk of cognitive decline among the general elderly population. This may have significant impact on public health. Furthermore, SHS exposure was evaluated by utilizing both self-reported data and the concentration of serum cotinine measured by ID HPLC-APCI MS/MS, which is a reliable biomarker for SHS exposure. The inclusion of serum cotinine concentration helps to minimize measurement errors and provide more accurate results regarding SHS. Nonetheless, there are certain limitations in the present study. Firstly, the cross-sectional design of the study makes it challenging to make a causal explanation of the results. Secondly, self-reported smoking among senior citizens can lead to random and systematic errors, as well as potential recall bias. Nevertheless, we mitigated this issue by incorporating serum cotinine as a biomarker for SHS exposure to ensure the reliability. Thirdly, although we adjusted for confounders as much as possible in the final model, we cannot rule out that there are still additional possible confounders.

## 5 Conclusion

Cognitive impairment in the elderly population was found to be associated with VDD and SHS has a synergistic effect with VDD. This effect was particularly pronounced in overweight and obese individuals. Our findings add new theoretical support for the potential risk factors of cognitive impairment due to living habits that can be modified, helping facilitate planning and guiding targeted strategies in the elderly such as stricter restrictions on smoking in the circumstance, ensuring sufficient vitamin D intake as well as sun exposure to prevent and control cognitive impairment in elderly. Furthermore, our study highlights the need for future research to investigate the underlying mechanisms of the combined effects of VDD and SHS on cognitive impairment in the elderly.

## Data Availability

The raw data supporting the conclusions of this article will be made available by the authors, without undue reservation.
