# Supplementary material for: A synergistic effect of secondhand smoke with vitamin D deficiency on cognitive impairment in older adults: a cross sectional study
Source: Front Nutr. 2025 Feb 11;12:1533193. doi: 10.3389/fnut.2025.1533193 (PMC11852840; doi:10.3389/fnut.2025.1533193)
Supplement: Supplementary file 1 [file Data_Sheet_1.pdf]

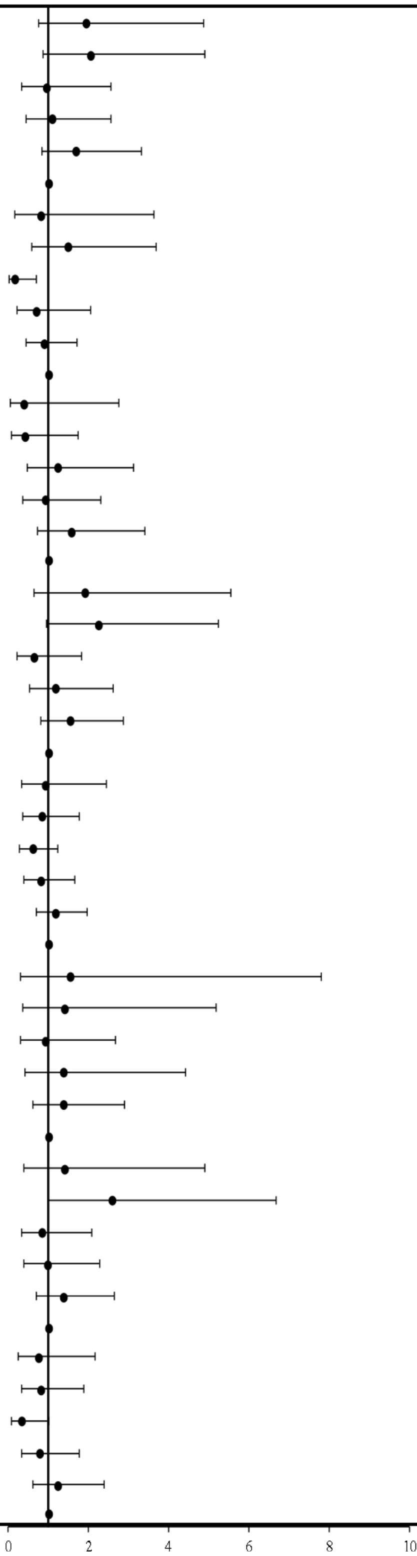

Subgroup			CERAD OR (95% CI)	P-value
**Age**	≥60 years	Deficiency*SHS	1.93(0.76, 4.87)	0.166
		Insufficiency*SHS	2.06(0.87, 4.90)	0.100
		Sufficiency*SHS	0.94(0.34, 2.58)	0.907
		Deficiency*non-SHS	1.10(0.47, 2.57)	0.822
		Insufficiency*non-SHS	1.67(0.84, 3.32)	0.142
		Sufficiency*non-SHS	Ref.	—
	≥70 years	Deficiency*SHS	0.82(0.18, 3.65)	0.793
		Insufficiency*SHS	1.49(0.60, 3.69)	0.387
		Sufficiency*SHS	0.15(0.03, 0.71)	0.017
		Deficiency*non-SHS	0.71(0.24, 2.07)	0.527
		Insufficiency*non-SHS	0.88(0.45, 1.73)	0.714
		Sufficiency*non-SHS	Ref.	—
	≥80 years	Deficiency*SHS	0.39(0.06, 2.76)	0.346
		Insufficiency*SHS	0.42(0.10, 1.74)	0.230
		Sufficiency*SHS	1.22(0.48, 3.13)	0.677
		Deficiency*non-SHS	0.91(0.36, 2.31)	0.834
		Insufficiency*non-SHS	1.58(0.74, 3.40)	0.238
		Sufficiency*non-SHS	Ref.	—
**Gender**	Male	Deficiency*SHS	1.90(0.65, 5.55)	0.244
		Insufficiency*SHS	2.25(0.97, 5.25)	0.060
		Sufficiency*SHS	0.65(0.23, 1.83)	0.418
		Deficiency*non-SHS	1.18(0.53, 2.63)	0.684
		Insufficiency*non-SHS	1.53(0.81, 2.88)	0.190
		Sufficiency*non-SHS	Ref.	—
	Female	Deficiency*SHS	0.92(0.35, 2.45)	0.871
		Insufficiency*SHS	0.83(0.38, 1.79)	0.630
		Sufficiency*SHS	0.61(0.30, 1.24)	0.172
		Deficiency*non-SHS	0.82(0.40, 1.68)	0.588
		Insufficiency*non-SHS	1.18(0.71, 1.97)	0.514
		Sufficiency*non-SHS	Ref.	—
**BMI**	< 25 kg/m^2	Deficiency*SHS	1.55(0.31, 7.80)	0.596
		Insufficiency*SHS	1.40(0.38, 5.19)	0.614
		Sufficiency*SHS	0.91(0.31, 2.67)	0.870
		Deficiency*non-SHS	1.36(0.42, 4.43)	0.614
		Insufficiency*non-SHS	1.36(0.64, 2.90)	0.427
		Sufficiency*non-SHS	Ref.	—
	25 to < 30 kg/m^2	Deficiency*SHS	1.40(0.40, 4.90)	0.599
		Insufficiency*SHS	2.58(1.00, 6.68)	0.050
		Sufficiency*SHS	0.85(0.35, 2.10)	0.728
		Deficiency*non-SHS	0.97(0.41, 2.30)	0.938
		Insufficiency*non-SHS	1.38(0.72, 2.64)	0.332
		Sufficiency*non-SHS	Ref.	—
	≥ 30 kg/m^2	Deficiency*SHS	0.76(0.27, 2.18)	0.614
		Insufficiency*SHS	0.81(0.34, 1.90)	0.625
		Sufficiency*SHS	0.32(0.10, 1.01)	0.051
		Deficiency*non-SHS	0.78(0.34, 1.77)	0.547
		Insufficiency*non-SHS	1.22(0.62, 2.39)	0.570
		Sufficiency*non-SHS	Ref.	—

Supplementary Figure 1. Subgroup analysis of the interaction of 25(OH)D and SHS on the risk of CERAD in the elderly, NHANES 2011-2014 (N=1446).

*: significant at $p < 0.05$. Abbreviations: CERAD, Consortium to Establish a Registry for Alzheimer's Disease; SHS, secondhand smoke; BMI, body mass index.

Subgroup		AFT	OR (95% CI)	P-value
**Age**	≥60 years	Deficiency*SHS	3.23(1.51, 6.93)*	0.003
		Insufficiency*SHS	1.46(0.66, 3.01)	0.348
		Sufficiency*SHS	0.49(0.18, 1.31)	0.155
		Deficiency*non-SHS	0.84(0.38, 1.88)	0.679
		Insufficiency*non-SHS	1.19(0.64, 2.20)	0.582
		Sufficiency*non-SHS	Ref.	—
	≥70 years	Deficiency*SHS	1.00(0.26, 3.86)	0.999
		Insufficiency*SHS	0.41(0.14, 1.15)	0.089
		Sufficiency*SHS	0.81(0.33, 2.02)	0.658
		Deficiency*non-SHS	0.72(0.26, 2.00)	0.562
		Insufficiency*non-SHS	0.96(0.50, 1.83)	0.899
		Sufficiency*non-SHS	Ref.	—
	≥80 years	Deficiency*SHS	3.21(0.50, 20.77)	0.222
		Insufficiency*SHS	3.06(0.77, 12.12)	0.112
		Sufficiency*SHS	2.21(0.88, 5.55)	0.093
		Deficiency*non-SHS	1.11(0.44, 2.80)	0.833
		Insufficiency*non-SHS	0.70(0.31, 1.57)	0.383
		Sufficiency*non-SHS	Ref.	—
**Gender**	Male	Deficiency*SHS	3.10(1.13, 8.49)*	0.028
		Insufficiency*SHS	1.10(0.45, 2.72)	0.837
		Sufficiency*SHS	2.11(0.87, 5.08)	0.097
		Deficiency*non-SHS	0.99(0.43, 2.28)	0.986
		Insufficiency*non-SHS	0.76(0.39, 1.50)	0.436
		Sufficiency*non-SHS	Ref.	—
	Female	Deficiency*SHS	2.21(1.06, 4.63)*	0.035
		Insufficiency*SHS	0.87(0.44, 1.74)	0.699
		Sufficiency*SHS	0.62(0.32, 1.20)	0.153
		Deficiency*non-SHS	0.82(0.42, 1.59)	0.562
		Insufficiency*non-SHS	1.15(0.72, 1.85)	0.560
		Sufficiency*non-SHS	Ref.	—
**BMI**	< 25 kg/m^2	Deficiency*SHS	1.72(0.42, 7.02)	0.449
		Insufficiency*SHS	0.68(0.20, 2.33)	0.544
		Sufficiency*SHS	1.28(0.52, 3.15)	0.590
		Deficiency*non-SHS	0.59(0.17, 2.08)	0.410
		Insufficiency*non-SHS	0.80(0.38, 1.67)	0.547
		Sufficiency*non-SHS	Ref.	—
	25 to < 30 kg/m^2	Deficiency*SHS	5.30(1.63, 17.24)*	0.006
		Insufficiency*SHS	1.40(0.53, 3.72)	0.496
		Sufficiency*SHS	0.84(0.34, 2.09)	0.711
		Deficiency*non-SHS	0.66(0.26, 1.72)	0.397
		Insufficiency*non-SHS	1.19(0.62, 2.31)	0.604
		Sufficiency*non-SHS	Ref.	—
	≥ 30 kg/m^2	Deficiency*SHS	2.41(1.04, 5.55)*	0.040
		Insufficiency*SHS	0.90(0.41, 1.99)	0.792
		Sufficiency*SHS	0.78(0.31, 1.96)	0.602
		Deficiency*non-SHS	1.18(0.57, 2.46)	0.659
		Insufficiency*non-SHS	0.93(0.49, 1.77)	0.825
		Sufficiency*non-SHS	Ref.	—

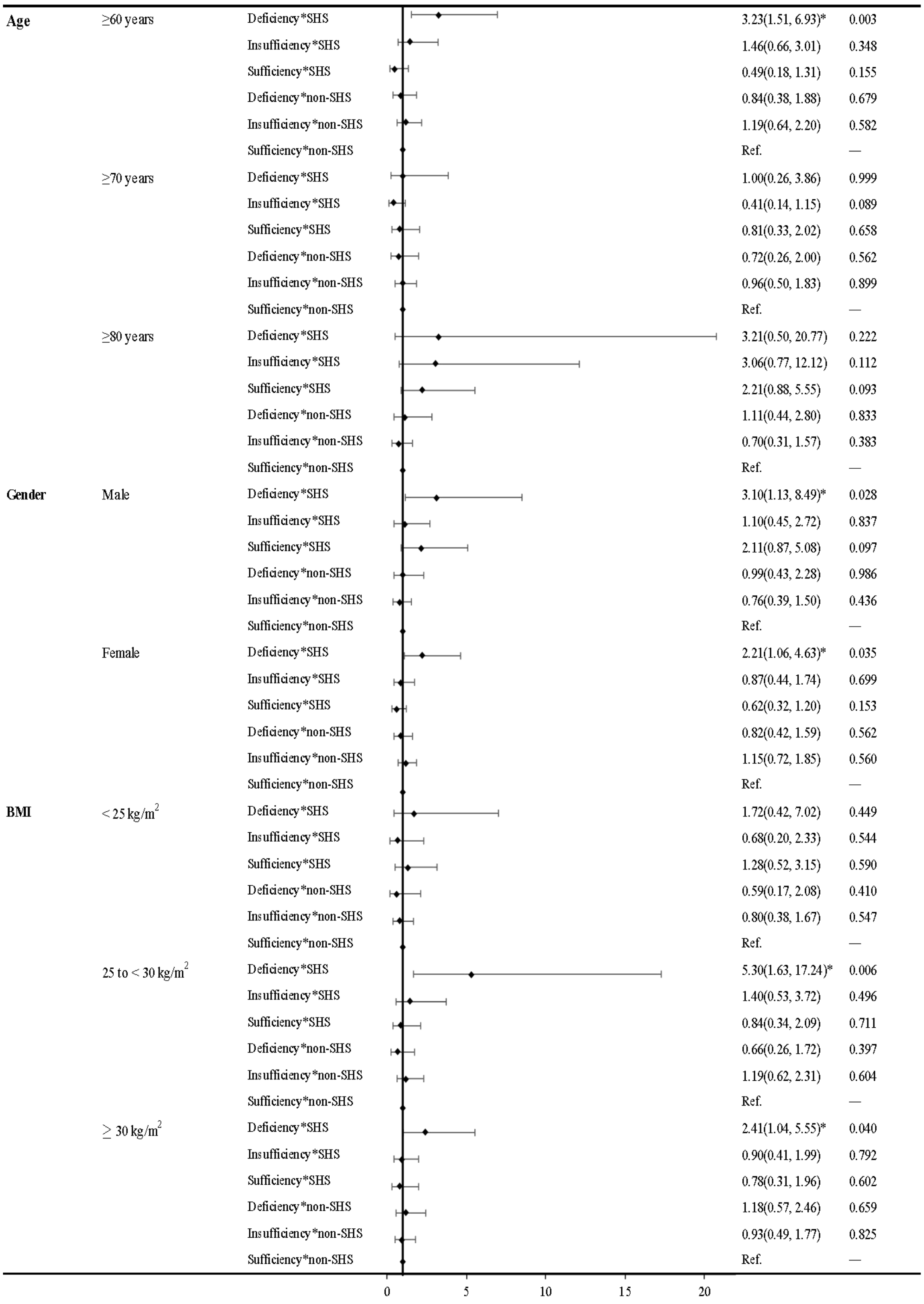

Supplementary Figure 2. Subgroup analysis of the interaction of 25(OH)D and SHS on the risk of AFT in the elderly, NHANES 2011-2014 (N=1446).

*: significant at $p < 0.05$. Abbreviations: AFT, the Animal Fluency test; SHS, secondhand smoke; BMI, body mass index.

Supplementary Table 1. Sensitivity analysis of cognitive function according to serum 25(OH)D concentrations and SHS

	CERAD			AFT			DSST		
	Crude Model	Model 1	Model 2	Crude Model	Model 1	Model 2	Crude Model	Model 1	Model 2
	OR (95% CI)	OR (95% CI)	OR (95% CI)	OR (95% CI)	OR (95% CI)	OR (95% CI)	OR (95% CI)	OR (95% CI)	OR (95% CI)
**25(OH)D level**									
<50 nmol/L	1.35(0.93, 1.95)	1.17(0.76, 1.79)	1.13(0.73, 1.77)	1.45(1.03, 2.04)*	1.40(0.94, 2.07)	1.30(0.86, 1.96)	1.70(1.20, 2.41)*	1.63(1.06, 2.51)*	1.68(1.07, 2.65)*
50-75 nmol/L	1.66(1.24, 2.21)*	1.45(1.04, 2.03)*	1.36(0.96, 1.93)	1.08(0.81, 1.44)	1.04(0.75, 1.44)	0.99(0.70, 1.39)	1.23(0.92, 1.65)	1.05(0.73, 1.51)	1.07(0.73, 1.57)
≥75 nmol/L	Ref.	Ref.	Ref.	Ref.	Ref.	Ref.	Ref.	Ref.	Ref.
**SHS**									
Yes	1.05(0.78, 1.42)	1.00(0.71, 1.41)	0.98(0.69, 1.40)	1.45(1.10, 1.93)*	1.30(0.94, 1.79)	1.28(0.92, 1.78)	1.44(1.08, 1.93)*	1.22(0.86,1.74)	1.15(0.79, 1.66)
No	Ref.	Ref.	Ref.	Ref.	Ref.	Ref.	Ref.	Ref.	Ref.

Model 1: adjusted for age, sex, race, education level, marital status, PIR

Model 2: Model 1+BMI, diabetes, stroke, asthma, congestive heart failure, alcohol use, and physical activity.

*: significant at $p < 0.05$

Abbreviations: CERAD, Consortium to Establish a Registry for Alzheimer's Disease; AFT, the Animal Fluency test; DSST, the Digit Symbol Substitution Test; SHS, secondhand smoke.

Supplementary Table 2. Sensitivity analysis of SHS and 25(OH)D synergistic effect on the risk of cognitive function

	CERAD			AFT			DSST		
	Crude Model OR (95% CI)	Model 1 OR (95% CI)	Model 2 OR (95% CI)	Crude Model OR (95% CI)	Model 1 OR (95% CI)	Model 2 OR (95% CI)	Crude Model OR (95% CI)	Model 1 OR (95% CI)	Model 2 OR (95% CI)
**Deficiency*SHS**	1.49(0.85, 2.63)	1.46(0.76, 2.82)	1.29(0.65, 2.58)	2.23(1.35, 3.71)*	2.57(1.46, 4.54)*	2.50(1.39, 4.50)*	2.58(1.54, 4.34)*	3.12(1.66, 5.87)*	3.11(1.60, 6.03)*
**Insufficiency*SHS**	1.72(1.09, 2.72)*	1.42(0.83, 2.42)	1.45(0.84, 2.51)	1.48(0.95, 2.32)	1.11(0.66, 1.87)	0.99(0.57, 1.73)	1.58(0.98, 2.23)	0.97(0.54, 1.73)	0.95(0.51, 1.76)
**Sufficiency*SHS**	0.80(0.48, 1.33)	0.72(0.41, 1.26)	0.64(0.36, 1.15)	1.14(0.73, 1.78)	0.93(0.56, 1.54)	0.94(0.56, 1.57)	1.22(0.77, 1.93)	0.89(0.51, 1.55)	0.75(0.41, 1.35)
**Deficiency*non-SHS**	1.20(0.77, 1.87)	0.94(0.56, 1.57)	0.93(0.55, 1.57)	1.17(0.77, 1.80)	0.93(0.57, 1.53)	0.83(0.49, 1.41)	1.43(0.93, 2.21)	1.06(0.62, 1.82)	1.06(0.60, 1.86)
**Insufficiency*non-SHS**	1.54(1.11, 2.15)*	1.34(0.91, 1.96)	1.16(0.78, 1.74)	0.98(0.70, 1.38)	0.99(0.68, 1.44)	0.96(0.65, 1.43)	1.19(0.84, 1.68)	1.04(0.68, 1.59)	1.02(0.66, 1.60)
**Sufficiency*non-SHS**	Ref.	Ref.	Ref.	Ref.	Ref.	Ref.	Ref.	Ref.	Ref.

Model 1: adjusted for age, sex, race, education level, marital status, PIR

Model 2: Model 1+BMI, diabetes, stroke, asthma, congestive heart failure, alcohol use, and physical activity.

*: significant at $p < 0.05$

Abbreviations: CERAD, Consortium to Establish a Registry for Alzheimer's Disease; AFT, the Animal Fluency test; DSST, the Digit Symbol Substitution Test; SHS, secondhand smoke.
